# Learning Impairment in Honey Bees Caused by Agricultural Spray Adjuvants

**DOI:** 10.1371/journal.pone.0040848

**Published:** 2012-07-16

**Authors:** Timothy J. Ciarlo, Christopher A. Mullin, James L. Frazier, Daniel R. Schmehl

**Affiliations:** Department of Entomology, The Pennsylvania State University, University Park, Pennsylvania, United States of America; Ghent University, Belgium

## Abstract

**Background:**

Spray adjuvants are often applied to crops in conjunction with agricultural pesticides in order to boost the efficacy of the active ingredient(s). The adjuvants themselves are largely assumed to be biologically inert and are therefore subject to minimal scrutiny and toxicological testing by regulatory agencies. Honey bees are exposed to a wide array of pesticides as they conduct normal foraging operations, meaning that they are likely exposed to spray adjuvants as well. It was previously unknown whether these agrochemicals have any deleterious effects on honey bee behavior.

**Methodology/Principal Findings:**

An improved, automated version of the proboscis extension reflex (PER) assay with a high degree of trial-to-trial reproducibility was used to measure the olfactory learning ability of honey bees treated orally with sublethal doses of the most widely used spray adjuvants on almonds in the Central Valley of California. Three different adjuvant classes (nonionic surfactants, crop oil concentrates, and organosilicone surfactants) were investigated in this study. Learning was impaired after ingestion of 20 µg organosilicone surfactant, indicating harmful effects on honey bees caused by agrochemicals previously believed to be innocuous. Organosilicones were more active than the nonionic adjuvants, while the crop oil concentrates were inactive. Ingestion was required for the tested adjuvant to have an effect on learning, as exposure via antennal contact only induced no level of impairment.

**Conclusions/Significance:**

A decrease in percent conditioned response after ingestion of organosilicone surfactants has been demonstrated here for the first time. Olfactory learning is important for foraging honey bees because it allows them to exploit the most productive floral resources in an area at any given time. Impairment of this learning ability may have serious implications for foraging efficiency at the colony level, as well as potentially many social interactions. Organosilicone spray adjuvants may therefore contribute to the ongoing global decline in honey bee health.

## Introduction

Colony Collapse Disorder (CCD) continues to be a major threat to honey bees worldwide. Colony losses in the USA have averaged 30%, 34%, 29%, 36%, and 32% in the winters of 2010–2011, 2009–2010, 2008–2009, 2007–2008, and 2006–2007, respectively [1]. These figures do not include non-winter colony losses that represent a broader decline of honey bees and other pollinators worldwide. At present, it is thought that multiple factors such as pathogens, parasites, malnutrition, and pesticide exposure have a role in CCD and the prevailing global diminishing of bees [2], [3].

The pesticide hypothesis has received considerable attention since the emergence of CCD in 2006. Foraging worker bees are exposed to pesticides in agro-ecosystems as they gather nectar and pollen from flowers, but only recently has the extent of this pesticide exposure been investigated. A comparative study of CCD-affected hives and healthy hives revealed the presence of 121 different pesticides and metabolites in 887 wax, pollen, and bee samples taken from managed hives across the U.S., with an average of 6 detections per sample [4]. However, no correlation was found between any one pesticide and CCD [3]–[5].

While considerable progress has been made with regard to surveying the prevalence of pesticide active ingredients within hives, virtually no work has been done to examine the safety of pesticide adjuvants that are either included in pesticide formulations (formulation adjuvants) or tank-mixed and sprayed along with the formulated product (spray adjuvants). Adjuvants are designed to boost the efficacy of sprayed fungicides, herbicides, and insecticides by improving spreading, wetting, penetration, reducing UV degradation, and/or reducing foaming and evaporation [6]–[9]. Spray tank adjuvants themselves are largely assumed to be biologically inert and are usually not included in risk assessment trials required to register a pesticide or its formulations [10], [11]. Moreover, the specific ingredients that make up spray adjuvants are considered trade secrets of the chemical companies that manufacture them and are therefore usually not disclosed [11], [12]. Given the fact that migratory honey bees are exposed to so many pesticides, and the fact that these adjuvants are sprayed in conjunction with pesticide formulations, it follows that foragers are likely exposed to adjuvants as well. The role of these agrochemicals in the ongoing investigation of CCD and their effects on the physiology/behavior of honey bees have therefore been overlooked up to this point.

Typical ecotoxicological testing for registering pesticides focuses on short-term assays designed to determine the LD-50 or LC-50 of a particular chemical in a population of test organisms. Consequently, many of the effects from chronic or sublethal exposure to pesticides are largely unexplored, in part because of the difficulty of conducting these tests [13]. Given the complex foraging, communicative, and navigational tasks honey bees must perform, sublethal effects of pesticides are especially important when compared to other, less sophisticated nontarget species [14].

The proboscis extension reflex (PER) assay used here is a well-established associative learning assay that effectively simulates the feeding events that occur at a flower in a controlled laboratory setting [15], [16]. Sublethal doses of pesticide active ingredients have been shown to impair this learning pathway in foraging honey bees. Decourtye et al. [17] treated honey bees orally with one of nine pesticide active ingredients and found that learning performances were reduced in four of the nine treatment groups. One of three doses of each active ingredient was administered, the highest of which was only 1/20^th^ of the 48-hour oral LD-50. Abramson et al. [18] found that tebufenozide and diflubenzuron, two insect growth regulator pesticides believed to be harmless to honey bees, both reduce learning performance in honey bees at sublethal levels. The relatively new neonicotinoid insecticides, in addition to being highly toxic to honey bees, also impair learning and memory at sublethal levels [13], [19]–[21]. This is particularly true for imidacloprid. These studies used the PER assay to examine only the effects of pesticide active ingredients, however. It is currently unknown what sublethal effects agricultural spray adjuvants have on foraging honey bees, specifically with respect to their learning abilities. Here we describe evidence of learning impairment in honey bees caused by a particular class of spray adjuvants known as organosilicones.

## Materials and Methods

### Adjuvants Evaluated

There are hundreds of agricultural spray adjuvants currently in use in the U.S., and the list of registered products is ever-expanding [22]. In the interest of practicality, it was necessary to limit experimental investigation to the adjuvants most frequently encountered by honey bees in commercial beekeeping operations. This study focused on the most commonly used spray adjuvants applied to almonds in the Central Valley of California for two main reasons:

1. The almond pollination in the Central Valley of California is the single largest pollination event in the world. Agrochemicals applied to these almond trees are therefore likely to have the greatest impact on honey bee health relative to other cropping systems. Furthermore, some pesticides – especially fungicides – are applied to almonds while the flowers are in bloom [23]. Given that foragers visit open flowers to collect pollen and nectar, this scenario represents the greatest potential hazard to foraging honey bees in terms of exposure to agrochemicals.
2. The state of California is unique among all other U.S. states in that it requires growers of all important food crops to report their pesticide use. Spray adjuvants are considered pesticides and must therefore be reported in the same way pesticide active ingredients are. This usage information is contained in the California Pesticide Information Portal (CalPIP), a database maintained by the California Department of Pesticide Regulations [24].

The CalPIP database was searched for usage information regarding three major classes of spray adjuvants (organosilicone adjuvants, nonionic surfactants, and crop oil concentrates) on almonds in the top almond producing counties in California in 2009 [25]. Among the organosilicones, Dyne-Amic was the most widely used adjuvant, followed by Syl-Tac and Sylgard 309 (Fig. S1). Activator 90, R-11, and Britz B-85 were the most heavily used nonionic surfactants (Fig. S2), while Penetrator, Crop Oil Concentrate, and Agri-Dex represented the most widely used crop oil concentrates (Fig. S3). These most frequently used adjuvants were included in this study. Britz B-85 could not be obtained, so Induce was used instead.

**Figure 1:**
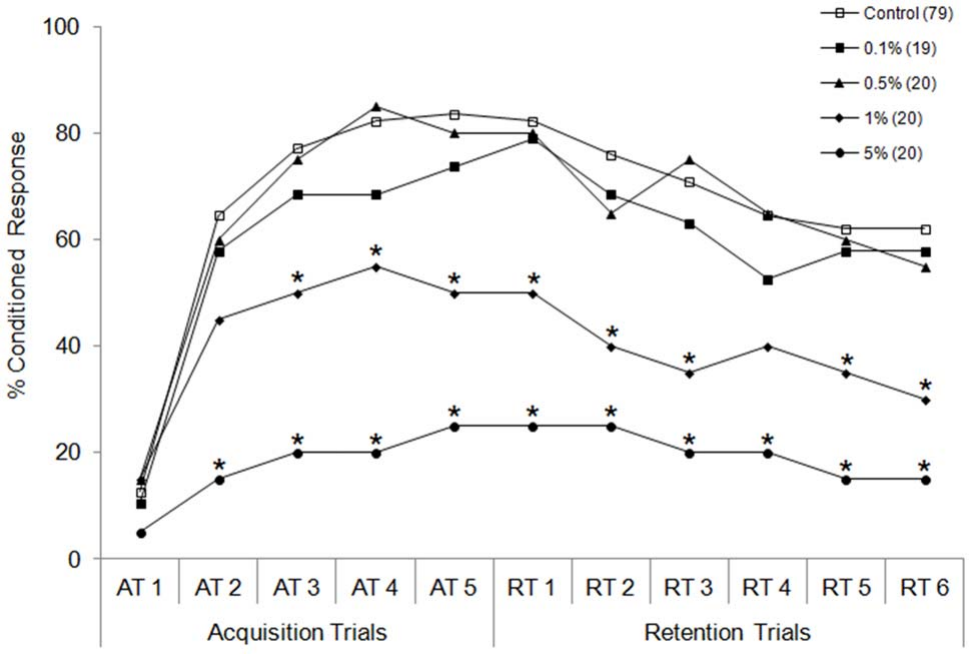
Learning performance of honey bees after antennal contact plus oral ingestion of Dyne-Amic. One of four concentrations of Dyne-Amic (5%, 1%, 0.5%, and 0.1%) in sucrose solution (50% w/v) was fed to bees 5 minutes prior to the first acquisition trial (AT1) for 2 seconds. Control bees were fed sucrose solution only. Percent conditioned response refers to the percentage of bees in each group that gave positive responses at each trial. The number of subjects in each group is indicated in parentheses. *P<0.05 (Fisher’s exact test) relative to the control.

Syl-Tac and R-11 were provided by Wilbur-Ellis (San Francisco, CA), and Sylgard 309 was provided by Dow-Corning (Midland, MI). Dyne-Amic, Silwet L-77, Induce, Penetrator, Agri-Dex, and Crop Oil Concentrate were provided by Helena (Collierville, TN), and Activator 90 was provided by Loveland (Greeley, CO). Technical grade imidacloprid (99.5% purity) was purchased from Chem Service (West Chester, PA). Imidacloprid is an active ingredient that reduces the learning ability of honey bees at sublethal doses [20] and was included to validate the methods used.

### Animals

Worker honey bees (*Apis mellifera* L.) were collected from one of two hives on the campus of Penn State University (University Park, PA) during the months of July to September of 2011. To ensure a homogenous age distribution among the test organisms, only approximately 2-week old post-nurse, pre-foraging ‘house’ bees from the uppermost box of each hive were selected. House bees are generally younger than foragers and are responsible for in-hive duties such as comb-building and handling of food resources [26]. Once collected, the bees were cold-anaesthetized for 3–4 minutes and individually harnessed in 2 cm lengths of 0.635 cm×0.432 cm (OD×ID) polyethylene tubing. Each tube was cut lengthwise so that it could be opened, and a semicircular piece of the tubing was cut from one end with a cork borer to allow the bee’s head and forelegs free range of motion (Fig. S4). Each bee’s wings extended out of the tube via the lengthwise slit and were wrapped with a small piece of parafilm to secure the bee in place. The harnessed bees were fed until satiated with a sucrose solution (50% w/v) and then starved for an 18-hour period at 25°C prior to the learning assays, aimed at establishing a uniform hunger level. We have found that house bees experience a lower incidence of mortality than foragers at the conclusion of this starvation period.

**Figure 2:**
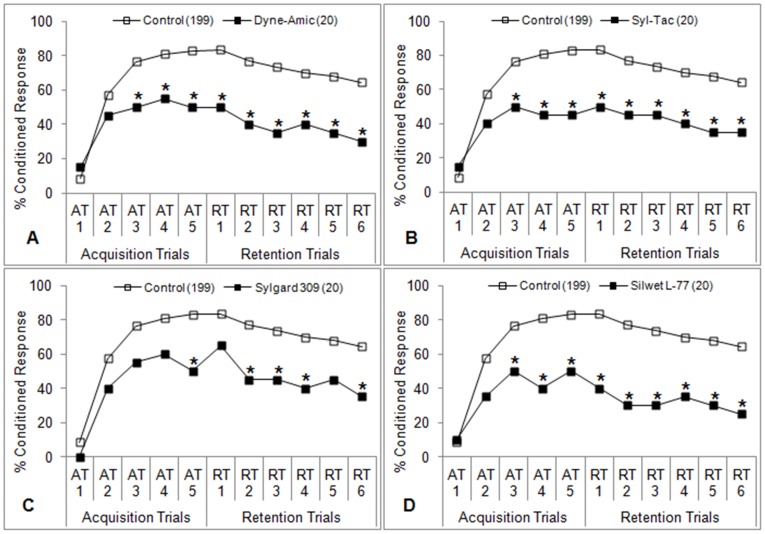
Learning performance of honey bees after antennal contact plus oral ingestion of organosilicone adjuvants. One of four different organosilicone adjuvants (1% v/v) in sucrose solution (50% w/v) was fed to bees 5 minutes prior to the first acquisition trial (AT1) for 2 seconds. Control bees were fed sucrose solution only. Percent conditioned response refers to the percentage of bees in each group that gave positive responses at each trial. The number of subjects in each group is indicated in parentheses. *P<0.05 (Fisher’s exact test) relative to the aggregate control.

**Figure 3:**
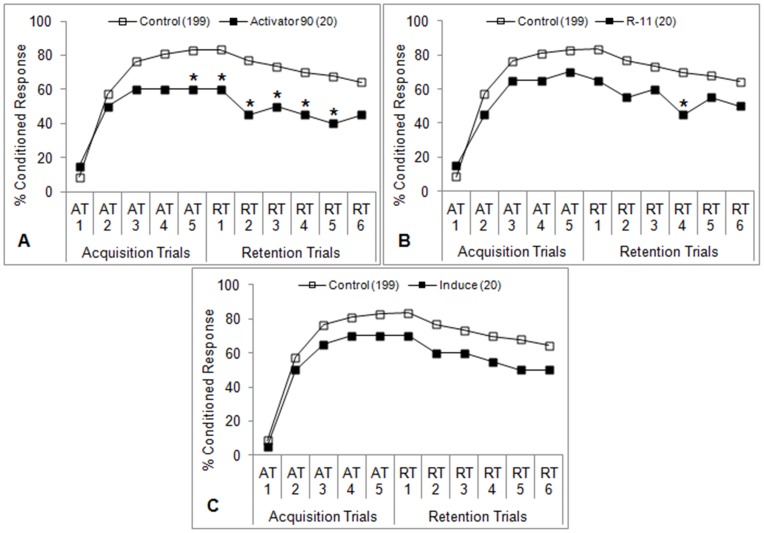
Learning performance of honey bees after antennal contact plus oral ingestion of nonionic surfactants. One of three different nonionic surfactants (1% v/v) in sucrose solution (50% w/v) was fed to bees 5 minutes prior to the first acquisition trial (AT1) for 2 seconds. Control bees were fed sucrose solution only. Percent conditioned response refers to the percentage of bees in each group that gave positive responses at each trial. The number of subjects in each group is indicated in parentheses. *P<0.05 (Fisher’s exact test) relative to the aggregate control.

**Figure 4:**
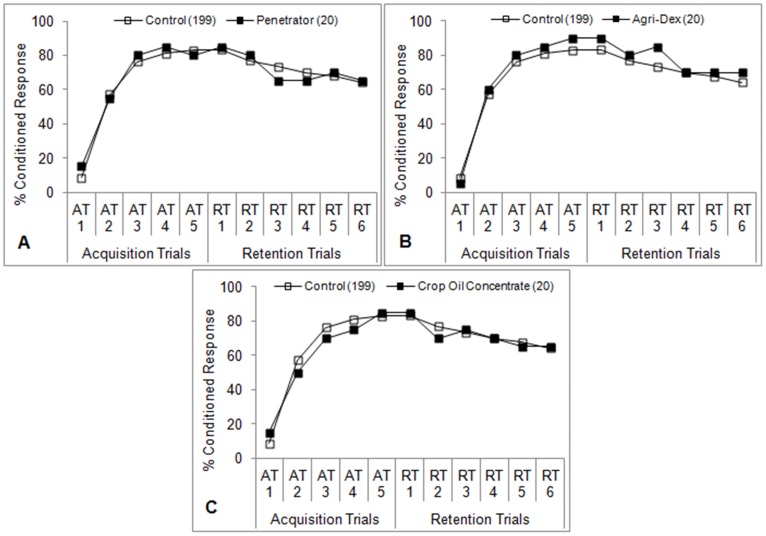
Learning performance of honey bees after antennal contact plus oral ingestion of crop oil concentrates. One of three different crop oil concentrates (1% v/v) in sucrose solution (50% w/v) was fed to bees 5 minutes prior to the first acquisition trial (AT1) for 2 seconds. Control bees were fed sucrose solution only. Percent conditioned response refers to the percentage of bees in each group that gave positive responses at each trial. The number of subjects in each group is indicated in parentheses. *P<0.05 (Fisher’s exact test) relative to the aggregate control.

### Exposure Protocols

After the 18-hour starvation period, honey bees were treated with one of the aforementioned adjuvants in one of three ways: oral exposure only (O), antennal contact only (A), and oral exposure plus antennal contact (A+O). For all treatment modalities, the adjuvant being tested was added to sucrose solution (50% w/v) to achieve an adjuvant concentration of 1% (v/v). In the ‘A+O’ experiments, this 1% adjuvant/sucrose solution was presented to the antennae of each bee in the treatment group via cotton swab. Upon contacting the saturated cotton swab, proboscis extension reflexively occurred, and each bee was allowed to feed on the adjuvant/sucrose solution for 2 seconds to simulate a nectar-feeding event at an adjuvant-sprayed flower (Fig. S5). In the ‘O’ experiments, a drop of sucrose solution (50% w/v) was presented to the antennae via a 16 gauge hypodermic needle to elicit proboscis extension such that the proboscis could not contact the drop. Instead, the proboscis (not antennae) was allowed to contact a cotton swab saturated with 1% adjuvant/sucrose solution for 2 seconds. In the ‘A’ experiments, the treatment method was simply reversed so that only the antennae contacted the adjuvant/sucrose solution. An equivalent number of control bees was used in each experiment. They were subjected to the same treatment method, but sucrose-only solution (50% w/v) was used in place of 1% adjuvant/sucrose solution. Treatment was administered 5 minutes prior to PER testing. In all cases, bees that did not extend their proboscis after antennal contact with either the 1% adjuvant/sucrose solution or sucrose-only solution (50% w/v) (depending on the experiment) were removed from the study and replaced with individuals that did have the reflex. ‘A’ and ‘O’ treatment protocols included only the most widely used adjuvant from each class (Dyne-Amic, Activator 90, and Penetrator) on almonds in the Central Valley of CA from 2005–2009.

**Figure 5:**
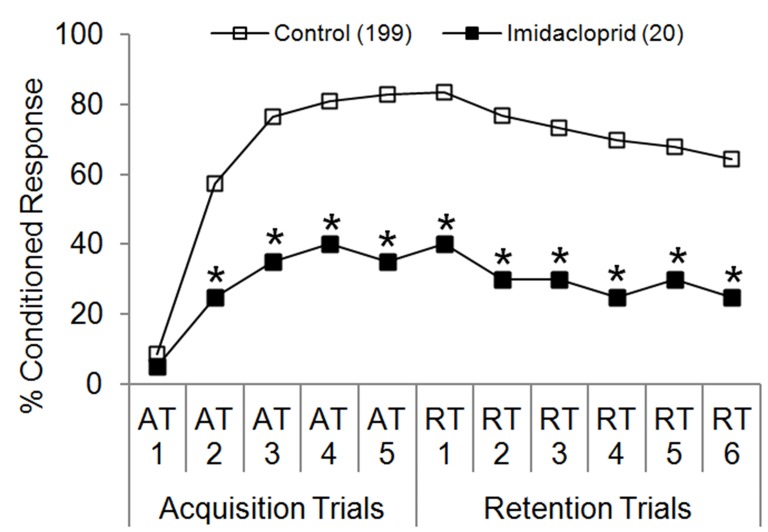
Learning performance of honey bees after antennal contact plus oral ingestion of 12 ng imidacloprid. Imidacloprid was fed to bees 5 minutes prior to the first acquisition trial (AT1). Control bees were fed sucrose solution only. Percent conditioned response refers to the percentage of bees in each group that gave positive responses at each trial. Number of subjects in each group is indicated in parentheses. *P<0.05 (Fisher’s exact test) relative to the aggregate control.

Technical grade imidacloprid was first dissolved in acetone and then diluted in sucrose solution (50% w/v) to achieve a concentration of 6.25 mg L^−1^. A 2 second feeding of this solution delivered an effective dose of 12 ng imidacloprid per bee, which has been shown to impair olfactory learning in honey bees [20]. The final concentration of acetone in the treatment solution was 1%. Control bees received sucrose solution (50% w/v, 1% acetone v/v).

**Figure 6:**
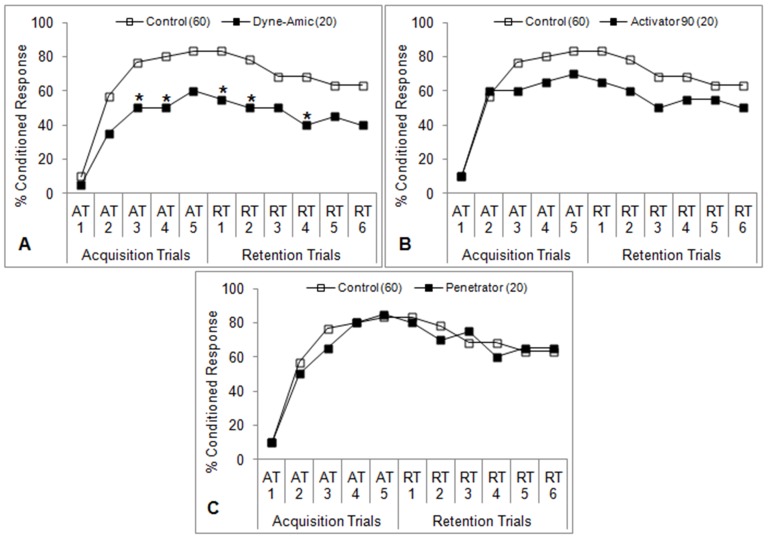
Learning performance of honey bees after oral ingestion (not antennal contact) of spray adjuvants. Bees were fed Dyne-Amic (**A**), Activator 90 (**B**), and Penetrator (**C**) (1% v/v for each adjuvant) 5 minutes prior to the first acquisition trial (AT1) for 2 seconds such that their antennae was not allowed to touch the adjuvant solution. Percent conditioned response refers to the percentage of bees in each group that gave positive responses at each trial. Number of subjects in each group is indicated in parentheses. *P<0.05 (Fisher’s exact test) relative to the aggregate control.

**Figure 7:**
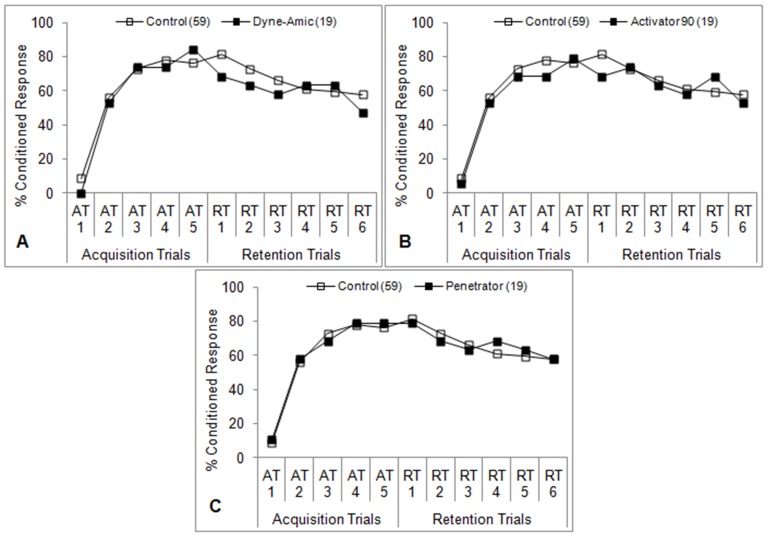
Learning performance of honey bees after antennal contact (not oral ingestion) with spray adjuvants. Bees’ antennae were touched with solutions of Dyne-Amic (**A**), Activator 90 (**B**), and Penetrator (**C**) (1% v/v for each adjuvant) 5 minutes prior to the first acquisition trial (AT1) for 2 seconds such that their mouthparts were not allowed to touch the adjuvant solution. Percent conditioned response refers to the percentage of bees in each group that gave positive responses at each trial. Number of subjects in each group is indicated in parentheses. *P<0.05 (Fisher’s exact test) relative to the aggregate control.

### Dyne-Amic Dose-PER Response

A dose-response study was undertaken to determine the lowest adjuvant concentration that leads to learning impairment. This concentration would then be used as a standard for all the adjuvants included in the three experimental protocols (A+O, A, and O). Dyne-Amic was added to sucrose solution (50% w/v), which was then administered to each bee in the treatment group according to the ‘A+O’ exposure protocol. 0.1%, 0.5%, 1.0%, and 5.0% were the four concentrations of Dyne-Amic tested.

**Figure 8:**
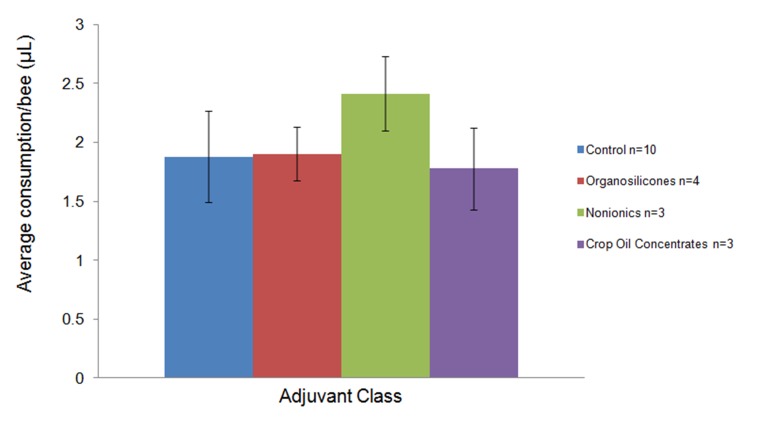
Amounts of treatment solution consumed per bee according to adjuvant class. Data for each individual adjuvant tested in the antennal contact plus oral ingestion experiments were treated as replicates according to class. Mean consumption amounts were 1.88, 1.90, 2.41, and 1.78 µL bee^−1^ for the control, organosilicone, nonionic, and crop oil concentrate classes, respectively. Error bars represent 1 S.D. of the mean. Amounts consumed per bee were not significantly different from the control (*P*<0.05, Student’s t-test).

### Learning Assays

The PER assays described here were adapted from Takeda [15] and Bitterman et al. [16]. The harnessed and treated honey bees were subjected to 5 acquisition trials (AT’s) followed by 6 retention trials (RT’s). Acquisition trials consisted of presentation of the conditioned stimulus (CS) plus the unconditioned stimulus (US), while retention trials consisted of presentation of the CS only. Sucrose solution (50% w/v) was used as the US, and the odor of a 1% solution of pure cinnamon oil (Now Foods, Bloomingdale, IL) in mineral oil was used as the CS. 500 µL of this cinnamon oil solution was placed into a vial with one air inlet tube and one outlet tube (Fig. S6). During each trial, a computer-controlled solenoid valve (3-Way MIV, The Lee Co., Essex, CT) operated by LabView software (v. 8.6, National Instruments, Austin, TX) directed a 5 second pulse of air into the bottom of the vial to produce a bubbling effect. The outlet tube then carried the injected air from the headspace of the vial and delivered it to the bee’s antennae. The bubbling ensured a consistent concentration of cinnamon odor in the 5 second pulse. A glass Pasteur pipette glued to the odor delivery assembly housed the outlet tube as well as a ‘non-pulse’ tube. This non-pulse tube carried a constant flow of non-odorous humidified Ultra Zero air (Fairless Hills, PA) from a gas cylinder to the bee whenever the 5 second CS pulse was not in effect. The solenoid valve redirected this airflow into the vial when the odor pulse command was given to produce the CS. Thus, a constant airflow was achieved (250 mL min^−1^ was used in this study) throughout each experiment, even at the beginning of the CS pulse. This constancy ensured that any positive response observed was due to the olfactory cue and not mechanosensory stimulation from a change in airflow.

A Pyrex 60° angle long-stem filtering funnel (VWR, Radnor, PA) with attached copper rings held a squad of 8 harnessed bees and was positioned in the arena with the aid of a manual micromanipulator (World Precision Instruments, Sarasota, FL). After each trial, the funnel remained in place but was rotated 45° to position the next bee in the squad in line with the air stream. After all 8 bees completed a single trial, the funnel was removed from the micromanipulator and another funnel introduced. The number of funnels used was dependent on the number of bees being tested. A separate glass funnel connected to a vacuum line, positioned directly behind the bee receiving the CS captured the exhaust once the CS odor passed over the antennae and removed it from the test arena. The automated odor delivery system described here is an improvement over previously described ‘manual’ methodologies [15], [16], [18], [27] that rely on a human operator to deliver a consistent pulse of odor in terms of duration and directional accuracy. Our PER method has a degree of reproducibility that has not been reported before.

During an acquisition trial, a 5 second CS pulse was delivered to the bee’s antennae. 3 seconds into this pulse, the US was presented via cotton swab first to the antennae to elicit proboscis extension, and then the proboscis itself. The bee was permitted to feed on the US for 1 second. Presentation of the US was therefore completed before the CS pulse was finished. Proboscis extension in the 3 second window before US presentation was seen as a positive response, and was recorded as a ‘yes’ or ‘no’ event. A 10 minute inter-trial interval was used in all experiments for both acquisition and retention trials.

During a retention trial, only the 5 second CS pulse was presented to the bee. Proboscis extension in this 5 second window was seen as a positive response. A 1 to 1.5 hour break occurred between the last acquisition trial and the first retention trial.

### Statistical Analysis

Comparisons of positive learning responses at each trial between control and treated groups were performed with the Fisher’s exact test. Student’s t-test was used to compare the mean consumption amounts of treatment solution per bee according to adjuvant class. A *P* value of less than 0.05 was considered significant. Data analyses were performed using Minitab statistical software (v. 14, State College, PA).

## Results

Control groups in each of the three treatment modalities (A+O, A, and O), including the acetone control group in the imidacloprid experiment, were not statistically different from each other and were pooled into an aggregate control group for each treatment modality. All comparisons between treated and control bees were made using these aggregate controls.

### Dyne-Amic Dose-PER Response

The olfactory learning performances of bees treated with the 4 concentrations of Dyne-Amic according to the ‘A+O’ protocol are shown in Fig. 1. Dyne-Amic did not cause a significant reduction in learning at concentrations of 0.1% and 0.5%. Significant learning impairment occurred in the 1.0% Dyne-Amic treated bees, beginning with AT3 (*P*<0.05, Fisher’s exact test). An even greater reduction in learning ability was seen in bees treated with 5.0% Dyne-Amic. Since 1.0% was the lowest concentration that significantly reduced learning, it was chosen as the standard concentration for the other adjuvants investigated here.

### Oral Exposure + Antennal Contact

The olfactory learning performances of bees treated with adjuvants according to the ‘A+O’ protocol are shown in Figs. 2, 3, 4. The organosilicone adjuvants (Dyne-Amic, Syl-Tac, and Silwet L-77) induced learning impairment beginning with the third acquisition trial (AT3) (Fig. 2) (*P*<0.05, Fisher’s exact test). The only other organosilicone tested, Sylgard 309, induced learning impairment beginning with AT5 (Fig. 2C) (*P*<0.05, Fisher’s exact test). Honey bees treated with the nonionic surfactant Activator 90 experienced a similar reduction in learning ability beginning at AT5 (Fig. 3A) (*P*<0.05, Fisher’s exact test). The other nonionic surfactants tested, R-11 and Induce, did not significantly impair learning (Fig. 3), although a difference was seen at the fourth retention trial (RT4) for R-11 (Fig. 3B) (*P*<0.05, Fisher’s exact test). No learning impairment was seen in bees treated with the three crop oil concentrates (Penetrator, Agri-Dex, and Crop Oil Concentrate) (Fig. 4).

Imidacloprid caused the most dramatic reduction in learning seen in this study, beginning with AT2 (Fig. 5) (*P*<0.05, Fisher’s exact test). No more than 40% of imidacloprid-treated bees gave positive responses at any one trial during PER testing.

### Oral Exposure Only

The olfactory learning performances of bees treated with the top adjuvant from each class according to the ‘O’ protocol are shown in Fig. 6. Bees treated with Dyne-Amic experienced a reduction in learning ability in line with those in the ‘A+O’ experiment, beginning with AT3 (Fig. 6A) (*P*<0.05, Fisher’s exact test). However, no statistical difference was seen at AT5, RT2, RT5, and RT6. Activator 90 did not significantly reduce learning, but there appears to be some degree of impairment (Fig. 6B). Penetrator did not cause any impact on learning ability (Fig. 6C). These results are similar to those observed in the ‘A+O’ experiments.

### Antennal Contact Only

When bees were treated with the top adjuvant from each class by antennal contact only (‘A’ protocol), no differences in learning ability were seen (Fig. 7). This holds true for Dyne-Amic, which induced a significant reduction in learning in both the ‘A+O’ and ‘O’ experiments. Thus, ingestion is required for the tested adjuvant to have an effect on learning. Direct action on the olfactory chemosensory cells to modify olfactory input to the learning association seems highly unlikely.

### Dose Consumed During Treatments

Mean consumption data according to class are shown in Fig. 8. These values were compared to the aggregate control (n = 10) and were not significantly different (*P*<0.05, Student’s t-test). The density of 50% sucrose solution (w/v) (ρ  = 1.1505 g mL^−1^) was used to convert mass to volume. 2 µL is the amount consumed per bee during a 2 second feeding. Given that the density of the nonionic and organosilicone surfactants is 1 g mL^−1^ or slightly greater than 1 g mL^−1^, a 2 second feeding of this solution delivered an effective dose of 20 µg adjuvant per bee. The average density of the crop oil concentrates was 0.875 g mL^−1^, meaning that a 2 second feeding delivered 17.5 µg per bee for these experiments.

## Discussion

Oral ingestion of 20 µg of the organosilicone adjuvants tested here (Dyne-Amic, Syl-Tac, Sylgard 309, and Silwet L-77) significantly reduces honey bees’ learning ability in the classical conditioning PER paradigm (Fig. 2). This is the first investigation into adverse effects on honey bees caused by supposedly inert agricultural spray adjuvants. Previous studies have found that sublethal doses of neurotoxic insecticides such as deltamethrin [17], [20], flucythrinate and cyfluthrin [28], thiamethoxam [21], and imidacloprid [19], [20], [29] impair learning ability in honey bees in a similar manner. Given that the neural connections in the honey bee brain, particularly those found in the mushroom bodies, play a large role in mediating associative learning and the formation of memory [30], [31], these results are not entirely surprising. Abramson et al. [18] showed that insecticides which are *not* neurotoxic, namely the insect growth regulators tebufenozide and diflubenzuron, can also interfere with associative learning in honey bees. Tebufenozide is an agonist of the molting hormone 20-hydroxyecdysone, causing development of a malformed cuticle when ingested by targeted insect larvae [32]. Diflubenzuron is a chitin inhibitor that disrupts synthesis of a new larval cuticle during molting [33]. The mechanism of action of organosilicone adjuvants with respect to learning impairment in honey bees remains unknown, but we also show that chemicals do not necessarily need to be classified as neurotoxic to have effects on learning and memory.

A series of initial experiments revealed 1% Dyne-Amic to be the lowest concentration that led to a significant decrease in learning ability following a 2 second feeding event (Fig. 1). Therefore, 1% was chosen as the standard concentration to be used for the other adjuvants included in the main part of this study. The fact that a clear dose-response was seen with Dyne-Amic further substantiates the evidence of learning impairment caused by organosilicone surfactants.

A comparable reduction in learning was not seen in bees treated with nonionic surfactants (Fig. 3), although percent conditioned responses were generally lower than those observed in the control bees. Activator 90 was the only nonionic surfactant to induce significantly lower positive learning responses at more than one trial. None of the crop oil concentrates tested caused significant reductions in learning (Fig. 4). The reasons as to why such a strong class effect was observed are unclear, but it may be due to the surfactant activity of each class. Organosilicones represent the newest class of agricultural surfactants and are known for their extreme spreading characteristics when added to aqueous solutions at very low concentrations [6], [34]. Most agrochemicals are sprayed onto leaf surfaces as aqueous solutions, and would ordinarily either bead up or be repelled outright by the waxy cuticle of a leaf. In either case, the total leaf area covered by the sprayed material is reduced, which in turn reduces the efficacy of the agrochemical [7]. Surfactants, when added at to aqueous solutions, reduce the surface tension of the solution, thereby allowing it to spread more readily on a nonpolar surface. Like all surfactants, each organosilicone molecule is composed of a hydrophilic group and a hydrophobic moiety that allow it to readily interact with both polar and nonpolar compounds. The ‘super-spreading’ ability of organosilicones is thought to be due to the siloxane backbone of the hydrophobic group, which allows the hydrophobe to be far more compact than that of conventional, carbon-based surfactants [6], [34]. The methyl (CH_3_) groups attached to each silicon atom are also more hydrophobic than the methylene (CH_2_) groups that comprise the hydrophobe portion of more conventional hydrocarbon surfactants [6], [7]. The end result is that organosilicones cause a greater reduction in surface tension than both nonionic surfactants and crop oil concentrates, making them the most potent surfactants available to growers even at lower concentrations. The mechanism of action within a honey bee that leads to learning impairment may be due to this extreme surfactant activity.

Organosilicone surfactants are also noted for their stomatal infiltration and penetrating characteristics. Most conventional herbicide adjuvants are not able to lower the surface tension of the sprayed solution to a point where stomatal infiltration is possible. Organosilicones can accomplish this in large part due to the compact nature of the siloxane hydrophobe, thereby increasing the efficacy of the herbicide with which they are sprayed [6], [7]. Perhaps of greater concern with respect to honey bee health is the super-penetrating aspect of organosilicones. Knight and Kirkwood [35] found that cuticular penetration of diflufenican in dicotyledonous weeds of winter cereals was enhanced when mixed with Silwet L-77 (1.0 g L^−1^). Again, this mechanism would increase the efficacy of herbicides by facilitating transport to their sites of action within the plant. Organosilicone adjuvants thus mediate both the mixing of hydrophobic pesticides with water to form solutions and the dissolution of hydrophobic cuticles and membranes to allow active ingredients to penetrate. It is unknown whether a similar phenomenon is taking place within the crop or midgut of honey bees.

In addition to being dependent on adjuvant class, learning impairment was also dependent on the route of exposure. No learning impairment was observed in the ‘A’ experiments (Fig. 7). In contrast, bees in ‘O’ experiments receiving the same adjuvants exhibited reduced learning ability in line with those in the ‘A+O’ experiments. This would suggest that the organosilicones (Dyne-Amic, at least) are not acting directly on the chemosensory neurons in the antennae, but rather at a systemic level after entering the crop. It is possible that they are acting on the gustatory receptors located on the proboscis itself, but this seems unlikely due to the fact that the gustatory receptors of the antennae were unaffected by any adjuvant.

Learning impairment is characterized by significantly lower percent conditioned responses in treated bees relative to control bees at any given trial during PER testing [13], [15], [16], [20], [28]. Each PER experiment consists of 5 acquisition trials followed by 6 retention trials. The acquisition trials begin 5 minutes after treatment with the adjuvant being tested and are designed to measure how well bees can form the association between CS and US. Retention trials begin 1 to 1.5 hours after the fifth acquisition trial and are designed to measure medium-term memory, or how well bees can recall a learned association. They are governed by different parameters and give different insights into the temporal mechanics of learning [14], [15], [16]. Lower percent conditioned responses during acquisition trials imply that the treatment has an immediate physiological effect on the bee, presumably in the central nervous system. It is important to note that a reduction in percent conditioned responses in the acquisition trials will have a concomitant effect on the retention trials for a given group of bees in a PER experiment. For instance, the retention trials for a particular treatment group may be significantly lower than the respective retention trials of the control group, but only because the acquisition of learning was already impaired. In other words, if there was no impairment during the acquisition trials, no significance would be seen in the retention trials. Indeed, this occurrence was seen in cases where organosilicones caused significant reductions in learning acquisition (Fig. 2). Bees in the organosilicone groups that were able to learn appeared to retain the memory of the association to the same extent as the control bees (i.e. the slope between RT1 and RT6 appears to be the same). It is therefore difficult to draw conclusions with regard to information obtained from the retention trials in these experiments. A reduction in percent conditioned responses during the retention trials, but not in the acquisition trials, would suggest a delayed effect of the treatment, as retention trials begin 2–3 hours after treatment. This scenario was not observed in any experiment conducted here, however.

A slight decrease in percent conditioned responses over the course of the retention trials is expected regardless of whether or not an adjuvant was administered. This decrease is likely due to habituation – a diminution of positive responses caused by repeated over-excitation of sensory neurons. Habituation rates between organosilicone-treated bees and control bees do not appear to be different based on the slope between RT1 and RT2.

The results described here have potentially serious implications for the future use and safety of spray adjuvants in agricultural systems. Foraging honey bees are exposed to numerous visual and olfactory cues each time they gather floral resources from a flower, and quickly learn to associate these stimuli with a reward of nectar or pollen [36]. This helps the colony as a unit rapidly switch from a less profitable nectar/pollen source to a more profitable one [26], [37]. The PER assay is a reliable and relatively easy way to simulate these events in the laboratory. A decrease in percent conditioned responses, which has been demonstrated here for the first time after ingestion of organosilicone surfactants, can be an indication of severe, colony-level impacts. The floral landscape is a dynamic one. The most profitable flowers in a given area change from day to day and can vary according to time of day [37], [38]. Weather and spatial distribution can also have huge impacts on floral resources. Optimal exploitation of the most profitable floral resources is vital to the success of a honey bee colony, and is dependent to a large extent on learning.

One of the hallmark symptoms of CCD and related bee decline syndromes is the rapid disappearance of adult bees away from the hive. This would seem to indicate that the causative agent is affecting the behavior of honey bees, not merely causing them to die. If that were the case, we would expect to see piles of dead bees around collapsed hives much like we see in cases of acute pesticide poisoning [3]. One hypothesis for the disappearance is that foragers are becoming disoriented while away on foraging trips and are unable to return to the hive. Chaffiol et al. [39] demonstrated that short-distance orientation performance of honey bees toward a floral compound is increased by prior conditioning of that floral compound in the PER paradigm. This study suggests that learning impairment detected in PER assays could be an indication of orientation impairment as well. This would need to be verified by field or semi-field studies. Nonetheless, other factors such as pathogens, parasites, malnutrition, and even old age contribute to bee disappearances away from the hive [2], [3], [26].

Given that the time between ingestion of the test adjuvant and AT1 is only 5 minutes, it is clear that effects on acquisition of learning are immediate. Thus, a forager ingesting nectar from an adjuvant-sprayed flower would be affected while it is still away from the hive. Disorientation at this time might prevent it from returning to the hive. The navigation system in honeybees relies on several mechanisms for orientation with respect to the hive and flowers. The sun’s azimuth as well as polarized light are the main cues foraging honey bees utilize to find previously learned foraging locations [26], [36], [37], [40], but these cues are not available during fully overcast weather conditions. Foraging, however, continues seemingly unhindered when the sky is overcast. Detection of the Earth’s magnetic field is one strategy for overcoming this problem [26], [37]. Honey bees are also able to learn the spatial arrangement of landmarks to orient themselves with respect to the hive. The acquisition of this spatial awareness is based on memory [41], [42]. It would be inappropriate to suggest that the PER assay is a valid measure of a honey bee’s navigational ability since it relies mostly on visual cues, but memory is important in both cases. It is not inconceivable that learning impairment as indicated by the PER assay could also be associated with an impairment of the backup navigation system of honey bees.

Without sampling for these adjuvants in the field, it is inappropriate to make conclusions about the concentration of these materials in the nectar of sprayed flowers (if they are present at all). One percent represents an appropriate starting point for the investigation of sublethal effects caused by spray adjuvants. Generally, spray adjuvants are added to tank-mixes at concentrations of less than 1%, but they may accumulate in nectar to concentrations higher than 1%, especially if multiple applications take place over a relatively short period of time. Moreover, a forager visiting multiple flowers that have been sprayed with an adjuvant/pesticide will receive a much larger overall dose than the dose investigated here, which was designed to simulate a single feeding event at a contaminated flower. Nectar loads of returning foragers typically weigh 25–40 mg [26]. A 2 second feeding of 50% sucrose solution (w/v) using the methods described here corresponds to roughly 2 mg, or 5–8% of an average nectar load. Additionally, the chemicals that make up spray adjuvants are often included in pesticide formulations as formulation adjuvants. These factors suggest that 1% is a conservative estimate of actual exposure. A detection protocol for spray adjuvants using LC-MS would also need to be developed, as these compounds – most notably the organosilicones – are notoriously difficult to detect using standard analytical methods.

Despite the widespread assumption that formulation ingredients and adjuvants are biologically inert, substantial evidence suggests that harm to non-target invertebrates is occurring. R-11, one of the nonionic surfactants investigated here, reduced the growth rate of *Daphnia pulex* at concentrations that would be expected after application near aquatic systems at recommended field rates [43]. Aqueous solutions of Silwet L-77, also investigated here, were toxic to two-spotted spider mites [44], Pacific spider mites, cotton aphids, western flower thrips, and grape mealybugs [45]), and fruit flies [46] at concentrations within the range of field application rates. The researchers in these studies even suggested that they might be valuable tools for control of these pests as they act in much the same manner as insecticidal soaps. Honey bees can also be affected by surfactants. A simple detergent solution has been shown to kill swarms of Africanized honey bees [47]. Goodwin and McBrydie [48] found that 4 out of 11 commercially available spray adjuvants (none studied here) were toxic to honey bees after topical application. Two of those 4 were toxic after oral application. While very few studies have examined the toxicity of adjuvants to honey bees, virtually none have been conducted to determine their potential sublethal effects.

### Conclusion

This study addresses the possibility that spray adjuvants impair olfactory learning, and thereby may contribute to disappearing honey bees that characterizes CCD and other bee decline syndromes. Traditional toxicological approaches that measure factors such as short-, or even long-term, mortality may fall short of accurately describing the effects of agrochemicals on the complex superorganism that is a honey bee colony. The PER assay is a well-established bioassay that measures the learning ability of honey bees, which is a vital component of effective foraging behavior. We have demonstrated here, for the first time, that agricultural spray adjuvants – and organosilicone surfactants in particular – do indeed cause significant learning impairment when ingested by honey bees. Their perceived status as ‘inert’ materials that can do no harm to biological organisms should be reconsidered. Field tests will need to be conducted to confirm these results on a colony-level, as events in the laboratory do not always translate to an organism’s natural setting. Further work to clarify specific ingredients in adjuvants responsible for the behavioral impacts are in progress.

## Supporting Information

Figure S1 **Amounts of organosilicone adjuvants applied to almonds in California’s Central Valley from 2005–2009.** Data was compiled from the California Department of Pesticide Regulation CalPIP database.
(TIF)

Figure S2 **Amounts of nonionic surfactants applied to almonds in California’s Central Valley from 2005–2009.** Data was compiled from the California Department of Pesticide Regulation CalPIP database.
(TIF)

Figure S3 **Amounts of crop oil concentrates applied to almonds in California’s Central Valley from 2005–2009.** Data was compiled from the California Department of Pesticide Regulation CalPIP database.
(TIF)

Figure S4 **Administration of conditioned stimulus (odor of 1% cinnamon oil) to harnessed bee showing proboscis extension.** Proboscis extension during the odor pulse but before the sucrose reward is given is recorded as a positive response and indicates that the bee has learned the association between conditioned and unconditioned stimuli.
(TIF)

Figure S5 **Administration of unconditioned stimulus (50% sucrose w/v).** The unconditioned stimulus is touched to the antennae first and then fed to bee for 1 second once the proboscis extends. The exhaust funnel that removes the conditioned stimulus odor from the test area can be seen directly behind the bee receiving the stimuli.
(TIF)

Figure S6 **Automated odor delivery apparatus showing 3-Way MIV solenoid valve.** The vial contains 500 µL of 1% cinnamon oil/mineral oil solution (v/v). A 5 second pulse of Ultra Zero air is directed into the vial to produce the conditioned stimulus.
(TIF)
