# Supplementary figures and images for: Learning Impairment in Honey Bees Caused by Agricultural Spray Adjuvants
Source: PLoS One. 2012 Jul 16;7(7):e40848. doi: 10.1371/journal.pone.0040848 (PMC3397935; doi:10.1371/journal.pone.0040848)

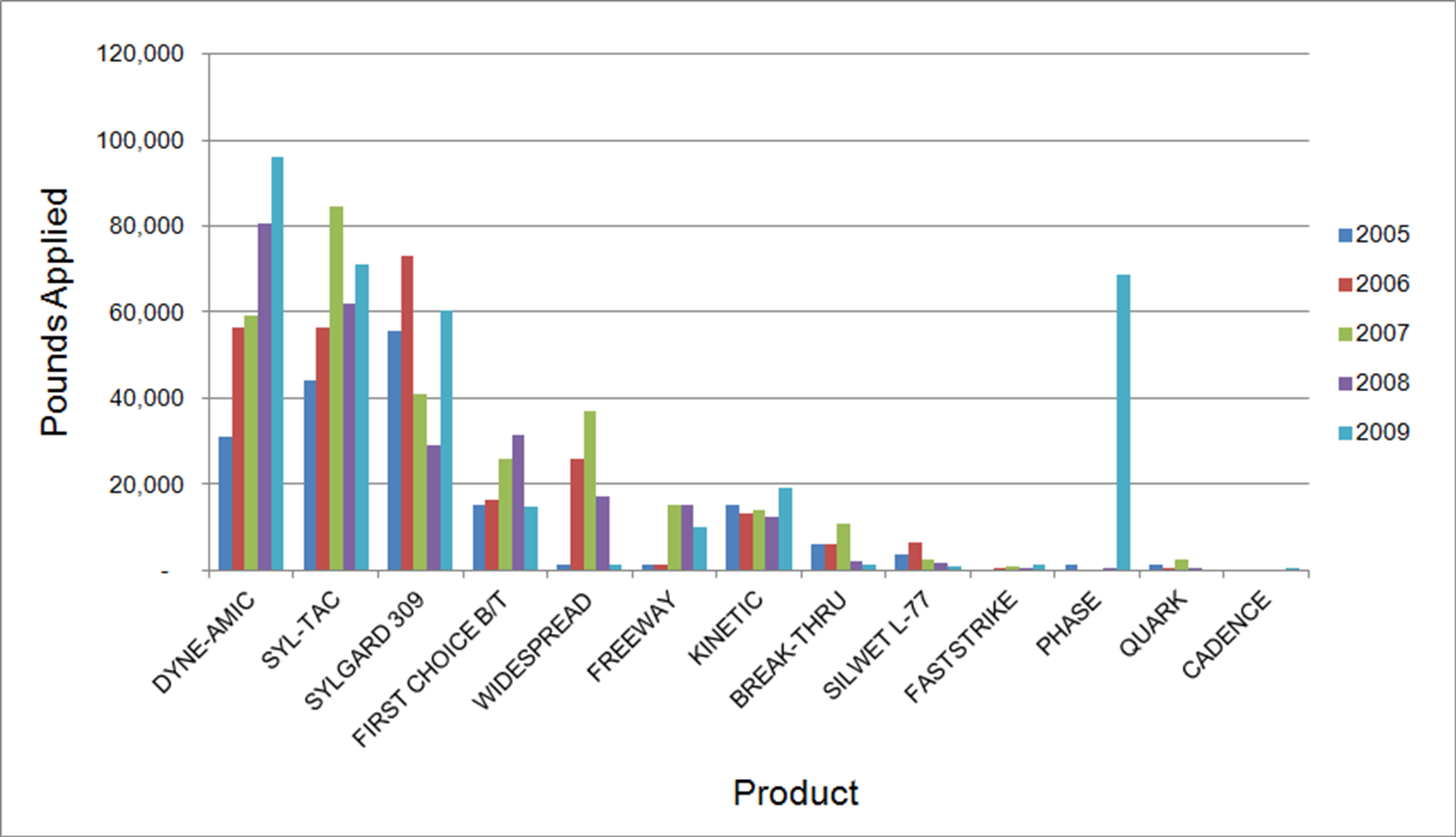

Supplement: Figure S1 — Amounts of organosilicone adjuvants applied to almonds in California’s Central Valley from 2005–2009. Data was compiled from the California Department of Pesticide Regulation CalPIP database. (TIF) [file pone.0040848.s001.tif]

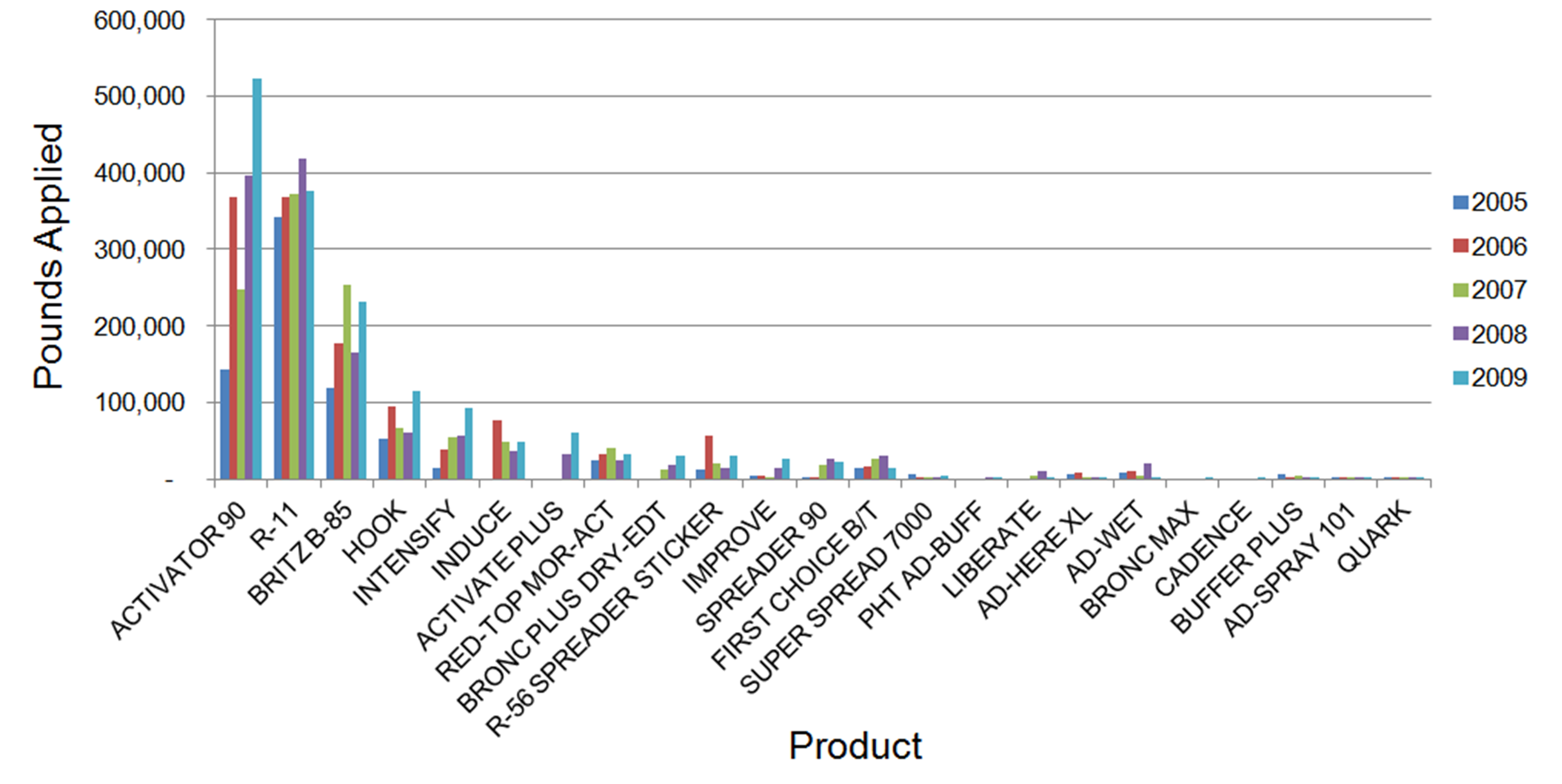

Supplement: Figure S2 — Amounts of nonionic surfactants applied to almonds in California’s Central Valley from 2005–2009. Data was compiled from the California Department of Pesticide Regulation CalPIP database. (TIF) [file pone.0040848.s002.tif]

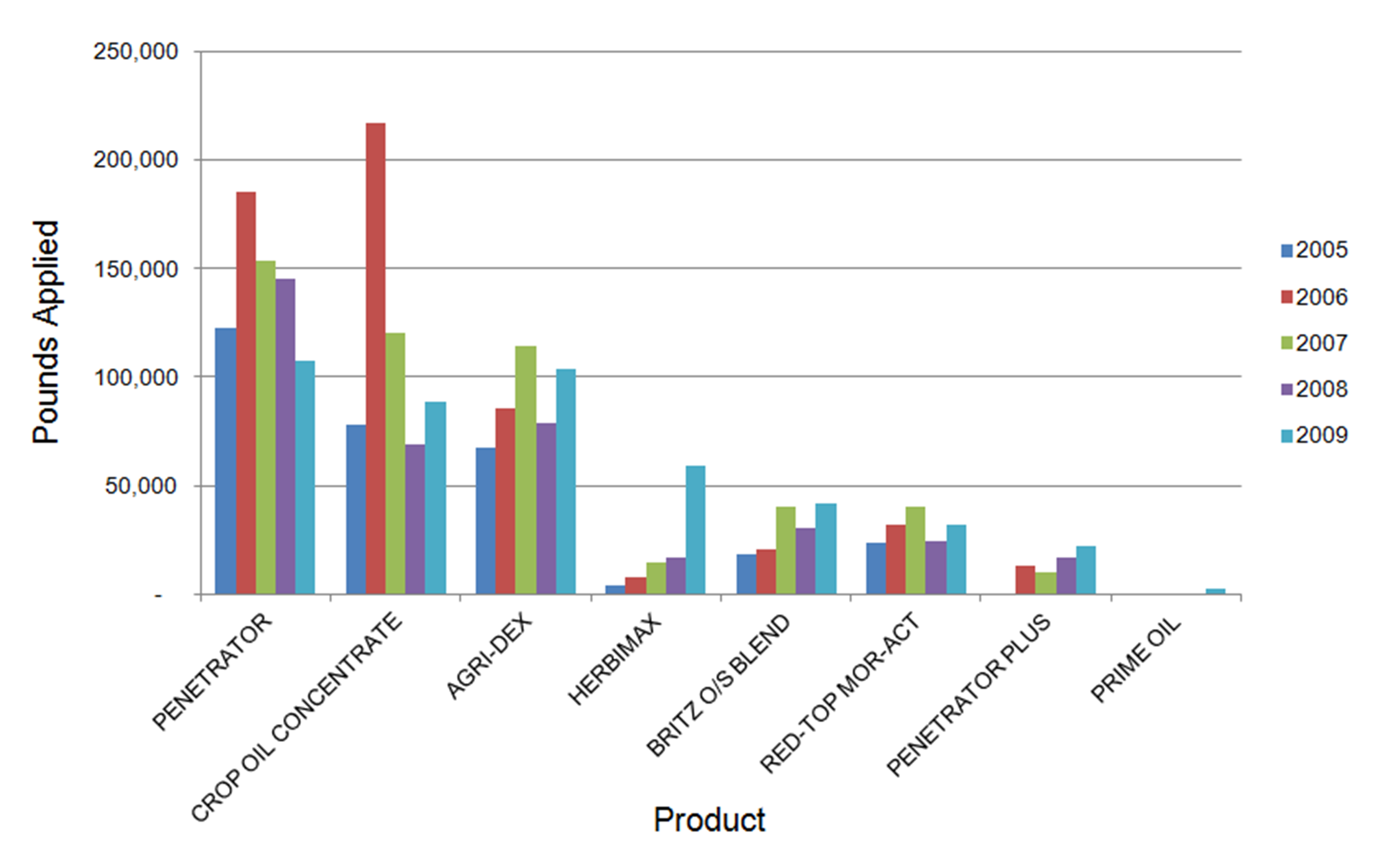

Supplement: Figure S3 — Amounts of crop oil concentrates applied to almonds in California’s Central Valley from 2005–2009. Data was compiled from the California Department of Pesticide Regulation CalPIP database. (TIF) [file pone.0040848.s003.tif]

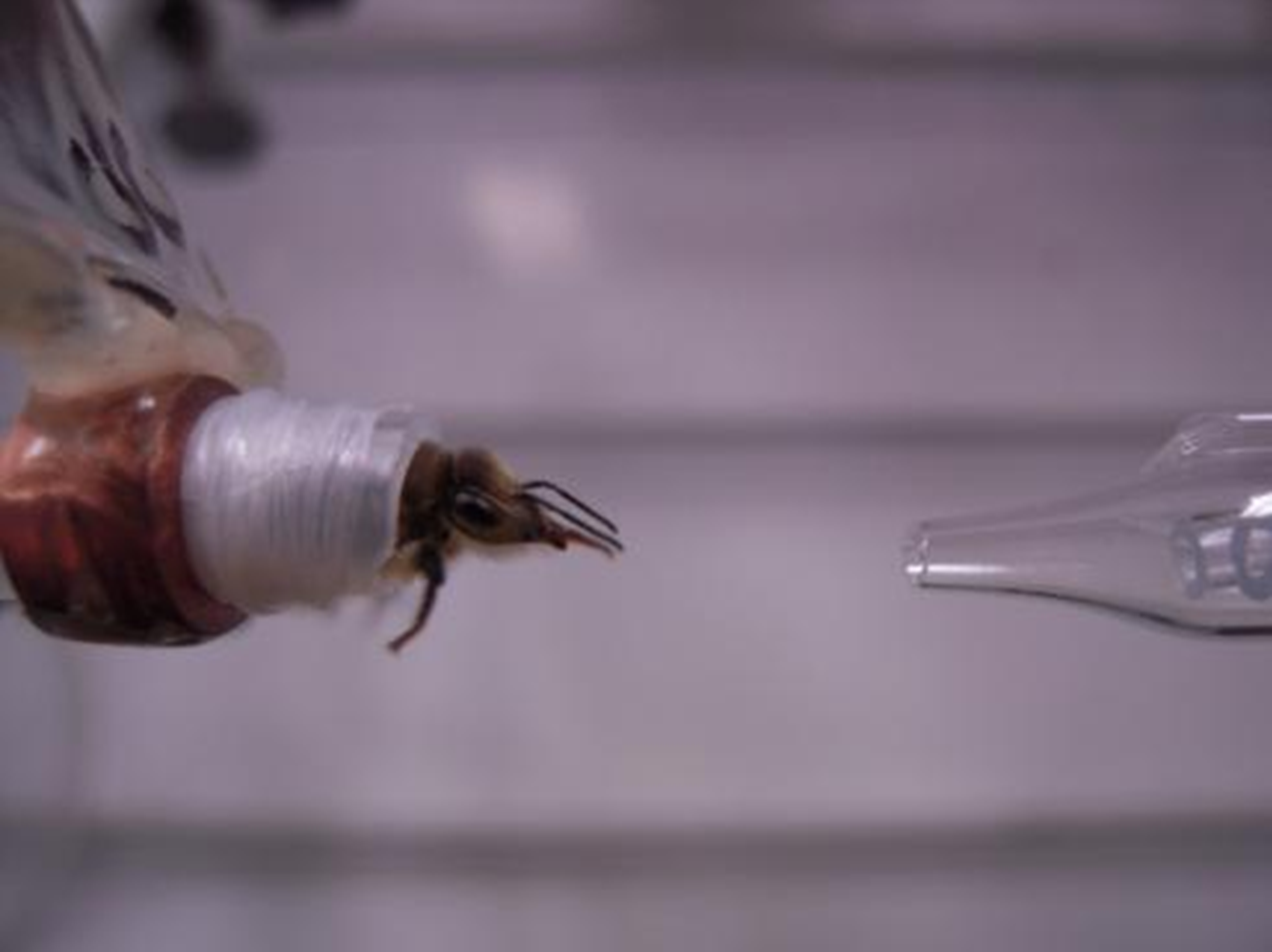

Supplement: Figure S4 — Administration of conditioned stimulus (odor of 1% cinnamon oil) to harnessed bee showing proboscis extension. Proboscis extension during the odor pulse but before the sucrose reward is given is recorded as a positive response and indicates that the bee has learned the association between conditioned and unconditioned stimuli. (TIF) [file pone.0040848.s004.tif]

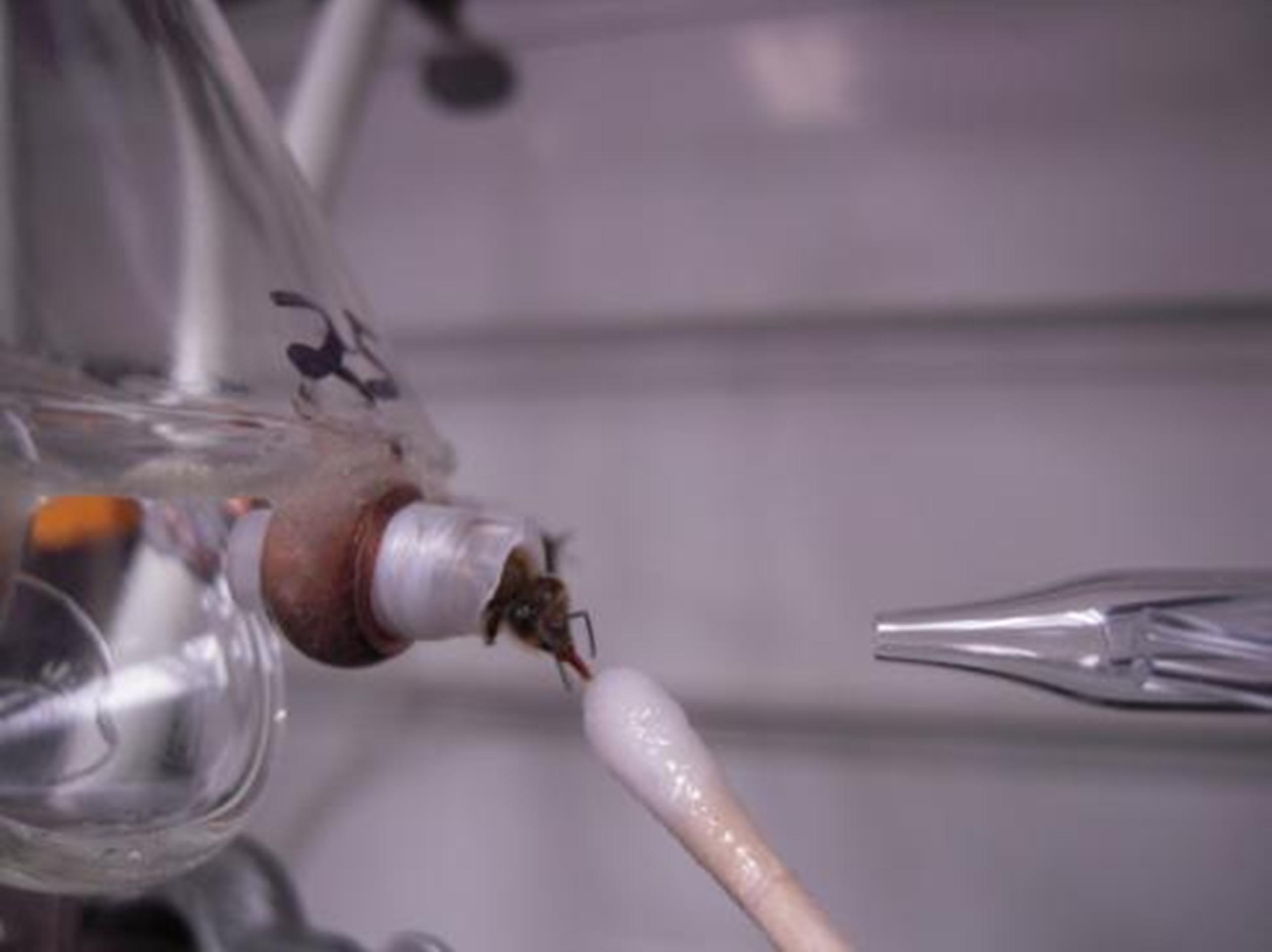

Supplement: Figure S5 — Administration of unconditioned stimulus (50% sucrose w/v). The unconditioned stimulus is touched to the antennae first and then fed to bee for 1 second once the proboscis extends. The exhaust funnel that removes the conditioned stimulus odor from the test area can be seen directly behind the bee receiving the stimuli. (TIF) [file pone.0040848.s005.tif]

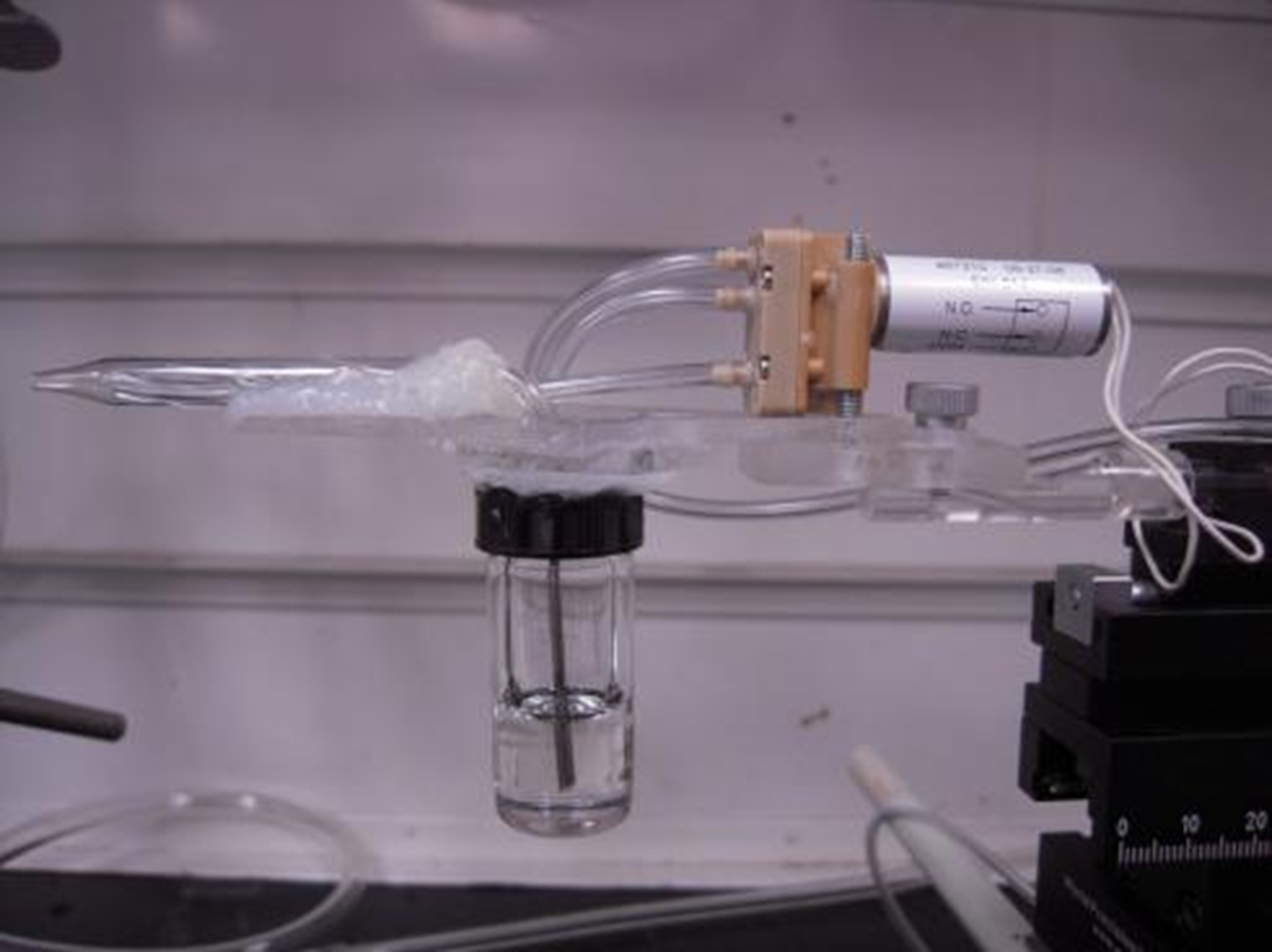

Supplement: Figure S6 — Automated odor delivery apparatus showing 3-Way MIV solenoid valve. The vial contains 500 µL of 1% cinnamon oil/mineral oil solution (v/v). A 5 second pulse of Ultra Zero air is directed into the vial to produce the conditioned stimulus. (TIF) [file pone.0040848.s006.tif]
